# Supplementary material for: A Global Screen for Assembly State Changes of the Mitotic Proteome by SEC-SWATH-MS
Source: Cell Syst. 2020 Feb 26;10(2):133–155.e6. doi: 10.1016/j.cels.2020.01.001 (PMC7042714; doi:10.1016/j.cels.2020.01.001)

O95295 | SNAPN_HUMAN | SNAPIN BLOC1S7 SNAP25BP SNAPAP
Monomer MW [kDa]: 14.874 Monomer expected elution fraction: 57

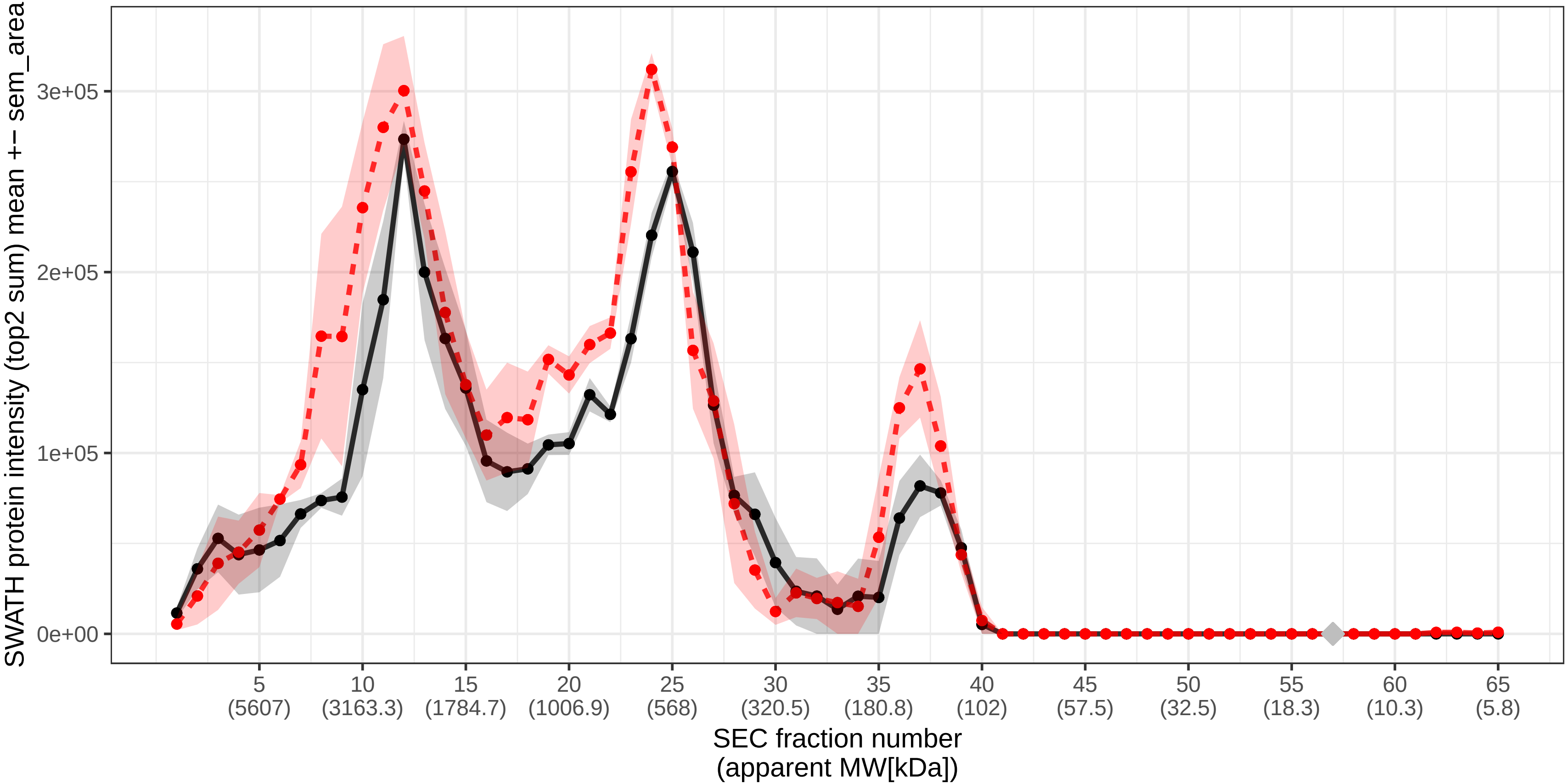

Supplement: Data S1. SEC-SWATH-MS Protein Chromatograms, Related to Figure 1 [file mmc6.zip › SECchrom_O95295_SNAPN_HUMAN_SNAPIN_BLOC1S7_SNAP25BP_SNAPAP.pdf]
